# Educational

**Published:** 1884-05

**Authors:** 


### EDUCATIONAL.

Below is given the list of graduates of each of the Dental Colleges of the United States, for the terms recently closed; with two or three exceptions, the reports of which have not come to hand. At each Commencement there was an address, or addresses, and an interesting time generally:

### Baltimore College of Dental Surgery.

FORTY-FOURTH ANNUAL COMMENCEMENT.

| NAME. | RESIDENCE. |
|---|---|
| R. A. Armstrong | Pennsylvania. |
| C. S. W. Baldwin | New Jersey. |
| F. C. Barlow | New Jersey. |
| A. R. Begun | Iowa. |
| Frank Boyette | North Carolina. |
| G. R. Brown | Maryland. |
| E. S. Carroll | Pennsylvania. |
| T. G. Carroll | Maryland. |
| Price Cheaney | Texas. |
| Wm. Crenshaw | Georgia. |
| A. S. Dabney | Kentucky. |
| Charles Diedel | Virginia. |
| M. H. Dodge | New York. |
| J. F. Dowsley | Massachusetts. |
| J. C. Duncan | Pennsylvania. |
| A. G. Finney | Maryland. |
| T. M. Fletcher | Arkansas. |
| A. H. Fox | New York. |
| F. P. Hamlet | New York. |
| H. W. Keidel | Texas. |
| Wm. T. Kelley | Maryland. |
| J. D. Lanier | Georgia. |
| I. E. Larrick | Virginia. |
| C. A. Meeker | New Jersey. |
| W. C. Mence | London, Eng. |
| Wm. A. Montelle | Maryland. |
| A. O'Daniel | North Carolina. |
| H. A. Parr | New York. |
| P. P. Ritter | Pennsylvania. |
| W. H. Ross | South Carolina. |
| T. H. Schaeffer | Maryland. |
| G. H. Slyfield | Illinois. |
| Dwight Smith | New York. |
| C. A. Timme | New Jersey. |
| F. A. Twitchell | Massachusetts. |
| J. A. Van Orsdel | Pennsylvania. |
| W. W. Walker | New York. |
| Whyte Ward | Louisiana. |
| J. A. Webb | Maryland. |
| G. W. Whitsett | North Carolina. |
| J. L. Williams | Connecticut. |
| T. P. Williams | Texas. |

### Ohio College of Dental Surgery.

THIRTY-EIGHTH ANNUAL COMMENCEMENT.

| NAME. | RESIDENCE. |
|---|---|
| B. E. Burger | Michigan. |
| Wm. L. Campbell | Illinois. |
| H. G. Chase | Indiana. |
| W. N. Dorward | Illinois. |
| G. B. Dillon | Illinois. |
| Theo. Erfort | Illinois. |
| E. E. Holloway | Ohio. |
| John W. Huston | Pennsylvania. |
| O. F. Lambertson | Nebraska. |
| A. H. Morey | Ohio. |
| Jesse Magee | Indiana. |
| J. C. McCormick | Ohio. |
| H. N. Renner | Ohio. |
| Carl H. Seeger | Wisconsin. |
| Oscar H. Simpson | Missouri. |
| Frank H. Smith | Pennsylvania. |
| B. Warder Shotwell | Ohio. |
| Edgar H. Wright | Pennsylvania. |
| Frank M. Williams | Colorado. |
| G. W. Williams | Kentucky. |
| W. J. Zinn | Kentucky. |

### Pennsylvania College of Dental Surgery.

TWENTY-EIGHTH ANNUAL COMMENCEMENT.

| NAME. | RESIDENCE. |
|---|---|
| Laurence C. Anderson | Mississippi. |
| George S. Andrews | New York. |
| Thomas Ashton | Pennsylvania. |
| William L. Bacon | Virginia. |
| Wilhelm Koehncke | Germany. |
| P. A. Ladmore, L.D.S. | England. |
| Hugh J. Laird | Pennsylvania. |
| Henry Leffmann, M.D. | Pennsylvania. |

| NAME. | RESIDENCE. |
|---|---|
| Maria K. Benkard | Germany. |
| E. R. C. Blackburn | Pennsylvania. |
| C. Curtis Coffee | Ohio. |
| George W. Cupit | Pennsylvania. |
| George Culbertson | Pennsylvania. |
| Marie D. C. Dalmer | Germany. |
| Vasco A. Da Silva | New York. |
| Fremont D. Davis. | Ohio. |
| Heinrich Deutschmann. | Germany. |
| William P. Dickinson. | Iowa. |
| J. Frank Dougherty. | Ohio. |
| Sophie E. Feltwell | Pennsylvania. |
| Alexander L. Foerster. | Pennsylvania. |
| August Gassner | Germany. |
| A. M. Green | Pennsylvania. |
| Hermann Gutzwiller | Switzerland. |
| A. W. Hafer | Pennsylvania. |
| James J. Hamilton | Pennsylvania. |
| Louis Haubeil | Germany. |
| Ferdinand Hegemann | Germany. |
| Edwin H. Hewit | Wisconsin. |
| Kurtz P. Hill. | Pennsylvania. |
| Pantaleon Hoyos | U.S.of Colombia. |
| Parker B. Hunt | Pennsylvania. |
| G. L. S. Jameson | Canada. |
| George A. Jarvis | Michigan. |
| Geo. Norman Johnson | New Hampshire. |
| Arthur S. Kniffin | New Jersey. |
| Alex. P. Long | Pennsylvania. |
| Victor Magliocco | Pennsylvania. |
| Henry Maser | Ohio. |
| C. H. M. Neall | Pennsylvania. |
| Nazario Noguera | South America. |
| Enrique Noguera | South America. |
| Harvey E. Nyer | Pennsylvania. |
| Juan Ph. Orozco | Central America |
| Pedro A. Palacio | Cuba. |
| Joseph A. Phillis | Pennsylvania. |
| Paul Pratz. | Germany. |
| Charlotte Renard | Germany. |
| Guy C. Rich | New York. |
| Nicolas Rocha | U.S.of Colombia. |
| J. Sabater Rivera | Porto Rico. |
| John Schembs | Pennsylvania. |
| Valentine Schmitt | Germany. |
| Courtland S. Service | Pennsylvania. |
| Marcus Sichel | Germany. |
| T. Frank Spencer | Rhode Island. |
| Herman Stocker | Switzerland. |
| Jose S. Tamayo | U.S.of Columbia. |
| Robert Addison Todd | Montana. |
| O. S. Watrous | Connecticut. |
| Charles Wetzel. | West Virginia. |
| Frederike Wiesner | Austria. |
| Marvin A. Wint | Pennsylvania. |
| William N. Wirt | Indiana. |

### Philadelphia Dental College.

### TWENTY-FIRST ANNUAL COMMENCEMENT.

| NAME. | RESIDENCE. |
|---|---|
| Elmer B. Abbey | Connecticut. |
| Thomas B. Arnold | Rhode Island. |
| J. Tenney Barker | Connecticut. |
| E. Bergstresser, M.D. | Pennsylvania. |
| Herbert A. Birdsall | New York. |
| Charles A. Bills | Maine. |
| John Darby Blakeslee | Ohio. |
| John H. Boozer | South Carolina. |
| Edw'd Brettschneider | Germany. |
| Philip H. Carlyon | Washington Ter. |
| Francis E. Cassidy | Massachusetts. |
| Hayes Agnew Clement. | Pennsylvania. |
| S.S. Costello | Maine. |
| Augustus R. Cooke | New York. |
| Norris R. Cox | Oregon. |
| Thomas A. Croaker | Nova Scotia. |
| William H. Davenport. | Massachusetts. |
| Horace G. Darling | Vermont. |
| Curtis L. Erwin | Pennsylvania. |
| John I. Evans | New Jersey. |
| George H. Fluck | Nova Scotia. |
| Edgar L. Gardiner | Rhode Island. |
| Matthew S. Gooding | New York. |
| Milton E. Gray | New York. |
| J. N. Harris | Maine. |
| George H. Hardisty | New York. |
| Merton R. Harned | Illinois. |
| William Evart Hall | New York. |
| Lloyd A. King | Kentucky. |
| Carl Knabe | Germany. |
| Jesse T. Krewson | Pennsylvania. |
| Samuel Lightcap | Pennsylvania. |
| William G. Long | Illinois. |
| William J. Magill | Connecticut. |
| T. R. Medd | Minnesota. |
| William H. Metcalf | New York. |
| Frank A. Monroe | New York. |
| George F. Nevius | Indiana. |
| Charles S. Rogers | Pennsylvania. |
| M. A. Richards | New York. |
| Hans Riegner | Germany. |
| Frederick O. Sawyer | Maine. |
| Bern'd D. Schlaudecker. | Pennsylvania. |
| Rafael S. Sevila | Mexico. |
| Louis N. Seymour | New York. |
| M. B. Smith | Pennsylvania. |
| William C. Stokes | New Jersey. |
| J. Harvey Stooksberry. | Ohio. |
| Randolph M. Stratton. | Pennsylvania. |
| Theodore Stanley, Jr | Missouri. |
| F. Tanzer, M.D | Austria. |
| W. Spencer Taylor | New York. |
| Edwin Ariel Thomas | Massachusetts. |
| James F. Thompson | Pennsylvania. |
| William Ray Tovell | Australia. |
| Ernest W. Tovell | Australia. |
| Alberto Vallarino | Panama. |
| Dan. J. Wait | Montana. |
| Clarendon P. Webster. | New Hampshire. |
| James L. Whinery | Iowa. |
| J. Harley Wood | New York. |
| William A. Woodward. | Pennsylvania. |
| Joseph J. Worsham | Georgia. |
| James F. Wright | Indiana |
| George H. Yates | New York. |

### New York College of Dentistry.

EIGHTEENTH ANNUAL COMMENCEMENT.

| NAME. | RESIDENCE. |
| --- | --- |
| John J. R. Beer | Switzerland. |
| Henry A. Benedict | New Jersey. |
| Cornelius E. Byrne | New York. |
| Charles L. Berger | New York. |
| Charles F. Burley | Connecticut. |
| Arthur J. Blersch | Germany. |
| William J. Caille, Jr. | New York. |
| Luis M. Clement | Panama. |
| Oliver L. Cole | New York. |
| Herman G. Hichborn | Maine. |
| Eugene L. Johnson | New York. |
| William R. Libbey | New York. |
| Manuel B. Lemus | U.S.of Colombia. |
| William Loewenthal | New Jersey. |
| Roderick M. Lowe | New York. |
| Charles W. Le Beau | Ohio. |
| William H. Milke | Pennsylvania. |
| Edward T. Mason | New York. |
| George J. Neish | Canada. |
| Edwin F. Normand | New York. |
| James J. Pruden | New York. |
| Edgar M. Davis | New York. |
| Francis Eschauzier | Spain. |
| Raymund Faquineto | Cuba. |
| Edmond A. Ferguson | New York. |
| Charles B. Gates | New York. |
| Friedrich Grau | Germany. |
| Edward L. Goodell | New York. |
| Stephen S. Hawley | Louisiana. |
| Arthur C. Hull | New Hampshire. |
| Walter E. Parrott | New York. |
| Eugenio E. Quesada | Cuba. |
| Jose B. Ross | Connecticut. |
| James A. Roberts | New Hampshire. |
| Edward J. Ranhoffer | New York. |
| Joseph J. Strohmeyer | New York. |
| Nathan Sanders | New York. |
| William M. Stanbrough | New York. |
| James W. Taylor | Uruguay, S. A. |
| Charles T. Van Derlip | New York. |
| Wesley Wait | New York. |
| Karl O. Wendler | Germany. |

### Missouri Dental College.

EIGHTEENTH ANNUAL COMMENCEMENT.

| NAME. | RESIDENCE. |
| --- | --- |
| Malcolm A. Bliss | Illinois. |
| Alfred E. Blake | California. |
| William O. Campbell | Missouri. |
| George S. Henderson | Missouri. |
| Warwick Winston. | Missouri. |
| William R. Wickersham | Missouri. |

### Dental College of the University of Michigan.

NINTH ANNUAL COMMENCEMENT.

| NAME. | RESIDENCE. |
| --- | --- |
| Thomas Allen | Michigan. |
| Rowland Wm. Bailey | Illinois. |
| Coldwell Corydon Beebe | Wisconsin. |
| William Cleland | Michigan. |
| Charles M. Cunningham | England. |
| Lyndall Llewllyn Davis | Michigan. |
| Louis Robert Esau. | Wisconsin. |
| Clarence James Hand | Texas. |
| Edson L. Hutchinson | Michigan. |
| Louis Manning James | Michigan. |
| Joel Dart Jones | Wisconsin. |
| Ezra Lincoln Kern | Ohio. |
| Donald Douglas Magill | Pennsylvania. |
| Louis James Mitchell | Ohio. |
| Minnie Ferris Mosher | Michigan. |
| George Eddy Sanders | Michigan. |
| Frank Germain Seger | Michigan. |
| John James Taylor | England. |
| Francis W. Temple | Michigan. |
| James Edward Winams | Pennsylvania. |
| * Francis E. Battershall | Ohio. |
| * Franklin R. Carson | Illinois. |
| * Alvin E. Unger | Pennsylvania. |
| * Chas. P. Weinrich | Illinois. |

### University of Tennessee—Dental Department.

SIXTH ANNUAL COMMENCEMENT.

| NAME. | RESIDENCE. |
| --- | --- |
| S. H. Baird | Alabama. |
| E. Beach | Tennessee. |
| J. T. Cazier | Tennessee. |
| E. O. Dickinson | Tennessee. |
| S. A. Hays | Tennessee. |
| T. W. Hogan | Georgia. |
| R. Scott Jamison | Mississippi. |
| H. R. Kline | Indiana. |
| M. D. Lanier | Georgia. |
| C. E. McConnell | Alabama. |
| S. G. Robinson | Alabama. |
| J. A. Wilson | Arkansas. |

* The four persons thus indicated above, will receive their degrees in June next.

### Vanderbilt University—Dental Department.

FIFTH ANNUAL COMMENCEMENT.

| NAME. | RESIDENCE. | NAME. | RESIDENCE. |
|---|---|---|---|
| Benjamin Green Blackwell | Texas. | Olympai Felix Gambati | Georgia. |
| Robert B. Chapman | Alabama. | William Hamilton McCurry | Georgia. |
| John Edward Frazier | Alabama. | Ferris Ewing Perkins | Georgia. |

### Indiana Dental College.

FIFTH ANNUAL COMMENCEMENT.

| NAME. | RESIDENCE. | NAME. | RESIDENCE. |
|---|---|---|---|
| John H. Adams | Indiana. | David C. Harold | Indiana. |
| Homer S. Ainsworth | Ohio. | G. Bennett Hough | Pennsylvania. |
| Frank C. Ayres | New York. | Merrit J. Kelley | Illinois. |
| Wilson M. Bayliss | Indiana. | Sanford M. Kennedy | Pennsylvania. |
| Jessie A. Bowman | Illinois. | Samuel A. Kiser | Ohio. |
| Herbert A. Chandler | Ohio. | Arkley D. Leach | Virginia. |
| Gerrit S. Clemens | Indiana. | Thomas H. Morgan | Indiana. |
| Cassius C. Curtis | Ohio. | Charles W. Myers | Indiana. |
| Richard Elson | Indiana. | Herbert W. Runyan | Michigan. |
| Ernest A. Gillet | Michigan. | Ozro W. Willis | Indiana. |

### University of Maryland—Department of Dental Surgery.

SECOND ANNUAL COMMENCEMENT.

| NAME. | RESIDENCE. | NAME. | RESIDENCE. |
|---|---|---|---|
| Theodore W. Albright | New York. | R. Dallas Kibler | Virginia. |
| James B. Bigham | South Carolina. | Wiley S. Killingsworth | South Carolina. |
| C. B. Blubaugh, M.D. | West Virginia. | Chas. J. Ladson | Dist. of Col. |
| Wilbur C. Bressler | Pennsylvania. | Clarence E. Lemley | Virginia. |
| Henry H. Boswell | Maryland. | Wm. Edward Lewis | Florida. |
| John H. Brown | Ohio. | Job B. Mallott | Pennsylvania. |
| George Buttler, Jr | New Jersey. | Charles H. McDowell | North Carolina. |
| John M. Comegys | Tennessee. | Semoney J. Minghin | West Virginia. |
| George V. Copp | Virginia. | S. Latimer Phillips | Virginia. |
| Almond J. Cutting | Massachusetts. | Geo. Edward Purnell | Maryland. |
| Isaac H. Davis | Maryland. | Nelson T. Shields | Texas. |
| Watson E. Dorchester | New York. | A. La F. Stratford | North Carolina. |
| Richard D. Evans | S. Wales, G. B. | Reading B. Swindel | North Carolina. |
| Claus Henry Fitler | Germany. | John E. Taggart | Vermont. |
| Nathan E. Foote | New York. | Robert R. Vaughan | Missouri. |
| Frank C. Gallup | Connecticut. | Richard van der Hoppe | Austria. |
| James Edwin Harris | Maryland. | Joseph T. Wayman | Virginia. |
| S. Dwight Hodge | Vermont. | Elmer J. Wisherd | Maryland. |

### University of Iowa—Dental Department.

SECOND ANNUAL COMMENCEMENT.

| NAME. | RESIDENCE. | NAME. | RESIDENCE. |
|---|---|---|---|
| J. O. Ball | Iowa. | F. R. Ross | Iowa. |
| J. H. Besore | Iowa. | M. H. Ross | Iowa. |
| F. C. Chamberlain | Iowa. | W. K. Sinton | Iowa. |
| Rufus Elmer | New Jersey. | J. W. Stark | Illinois. |
| T. M. Hallet | Iowa. | R. H. Tullis | Ohio. |
| S. G. Hill | Iowa. | W. H. Walker | Iowa. |
| S. W. Hill | Iowa. | | |

